# Effects of Pain Neuroscience Education Combined with Lumbar Stabilization Exercise on Strength and Pain in Patients with Chronic Low Back Pain: Randomized Controlled Trial

**DOI:** 10.3390/jpm12020303

**Published:** 2022-02-17

**Authors:** Ki-Sang Kim, Jungae An, Ju-O Kim, Mi-Young Lee, Byoung-Hee Lee

**Affiliations:** 1 Graduate School of Physical Therapy, Sahmyook University, Seoul 01795, Korea; kgs4590@naver.com (K.-S.K.); jungaean@hotmail.com (J.A.); k_juo@naver.com (J.-O.K.); 2 Department of Physical Therapy, Sahmyook University, Seoul 01795, Korea; mylee@syu.ac.kr

**Keywords:** neuroscience, patient education, muscle strength, pain

## Abstract

Chronic low back pain that lasts more than 12 weeks causes mental and physical distress. This study investigated the effects of pain neuroscience education combined with lumbar stabilization exercises on strength, pain, flexibility, and activity disorder index in female patients with chronic low back pain. Thirty-five female patients with chronic low back pain were randomly divided into two groups: the pain neuroscience education (PNE) combined with lumbar stabilization exercises (LSEs) group (*n* = 18, experimental group) and the lumbar stabilization exercises alone group (*n* = 17, control group). The experimental group underwent PNE combined with LSEs for 30 min per session, twice per week for 8 weeks, and the control group underwent LSEs only. The primary outcomes were strength (sit-up and back-up movements), Numerical Pain Rating Scale (NPRS), Korean Pain Catastrophizing Scale (K-PCS), and Tampa Scale of Kinesio-phobia-11 (TSK-11) for pain. The secondary outcomes were modified–modified Schober’s test (MMST) and finger to floor test (FFT) for flexibility and activity disorder (Roland–Morris Disability Questionnaire index). A significant difference was observed in the primary outcomes after intervention in the abdominal muscle strength (group difference, mean, −7.50; 95% CI, −9.111 to –5.889, F = 9.598; ANCOVA *p* = 0.005), the back muscle strength (group difference, mean, −9.722; 95% CI, −10.877 to –8.568, F = 7.102; ANCOVA *p* = 0.014), the NPRS (group difference, mean, 1.89; 95% CI,1.65 to 2.12, F = 24.286; ANCOVA *p* < 0.001), K-PCS (group difference, mean, 7.89; 95% CI, 7.02 to 8.76, F = 11.558; ANCOVA *p* = 0.003), and TSK-11 (group difference, mean, 16.79; 95% CI, 13.99 to 19.59, F = 13.179; ANCOVA *p* = 0.014) for pain. In the secondary outcomes, there was a significant difference in the FFT (group difference, mean, −0.66; 95%CI, −0.99 to −0.33, F = 4.327; ANCOVA *p* = 0.049), whereas the difference in flexibility (MMST) and activity disorder index of the secondary outcomes did not reach significance. Therefore, this study confirmed that PNE combined with LSEs is an effective intervention compared to LSE alone in improving muscle strength and pain in female patients with chronic low back pain.

## 1. Introduction

Low back pain is a common condition that affects the lives of many people, leading to high treatment costs, sick leave, and pain. It is the cause of one-fifth of all hospital visits and >80% of adults experience low back pain more than once in their lifetime [1]. Around 5~10% of low back pain progresses to chronic low back pain, mainly in women older than 40 [2,3].

Chronic low back pain usually refers to pain lasting more than 12 weeks and is associated with physical pain and fatigue from daily activities; it causes mental distress such as stress, depression, anxiety, impaired performance, and sleep disorders [4]. The causes of low back pain include changes in lifestyle habits, changes in the work environment, lack of exercise among modern people, and muscle weakness and decreased flexibility due to imbalance of the muscles around the lumbar region [5]. Among the epidemiological causes of low back pain, the most important is spinal instability [6], which causes pain, decreased endurance, decreased flexibility, and limitation in the range of motion of the lower back [7], and progresses to a chronic state. As the cross-sectional area of the muscles around the spine decreases, irreversible atrophy occurs.

The symptoms of low back pain are typical pain sensations in the lower lumbar region that occur frequently, such as dull pain, sharp pain, and aching pain [8]. When low back pain occurs, the sensitivity of the body increases [9], and its symptoms decrease the overall body activity owing to pain sensation, structural damage, and inhibition of the reflex contractile mechanisms of muscles. This leads to atrophy and muscle weakness, which worsen back pain and cause secondary spinal damage and physical disability [10].

Low back pain is treated using the Mulligan technique [11], manual traction with orthodontic treatment [12], thermal therapy and electrotherapy [13], and medications [14]. Physical inactivity negatively affects recovery from chronic low back pain, whereas exercise such as lumbar stabilization exercises (LSEs) has an effect in reducing pain and improving disability [14,15]. At present, LSEs are considered effective for improving health in patients with chronic low back pain, increasing the stabilization of the spine by training the movement pattern of the muscles [16], thus considerably reducing back pain. In addition, LSEs, which play an important role in providing dynamic stability to spinal segments, are useful for reducing dysfunction due to instability of the spine by strengthening the local muscle groups located in the deep trunk [6].

Recent low back pain is related not only to simple pain or dysfunction but also to psychosocial problems such as depression and poor quality of life [17]. Various physiological and psychological factors can be proposed as causes, and one possible cause is a direct injury [18]. Patients with chronic low back pain may experience psychological anxiety and depression due to physical and psychological stress resulting from limitations in physical activity [19]. Dysfunction in patients with chronic low back pain is highly associated with fear of pain and fear of movement. Pain neuroscience education (PNE) is an educational approach that deals with the nervous system in general and the physiology of the pain system, and has been conducted in patients with chronic musculoskeletal disorders such as chronic low back pain to address their thoughts and attitudes about pain for clinical effectiveness [20,21]. PNE does not solely focus on histopathology, but also on redefining the concept of pain by providing information about neurobiological and physiological processes based on the patient’s pain experience. Such attempts at reconceptualization help in understanding that pain and tissue damage are different concepts [22,23]. 

Although several studies have shown that the changes and improvements in functional and symptomatic outcomes with physical therapy and the application of PNE are effective in reducing pain and improving dysfunction [24,25], high-quality studies on PNE and exercise combinations for chronic low back pain are still needed because of the diversity of interventional exercise types, PNE application methods, and participants in previous studies [26,27].

Therefore, the purpose of this study was to compare the effects of PNE combined with LSEs on muscle strength, pain, flexibility, and activity disorder index in female patients with chronic low back pain. 

## 2. Materials and Methods

### 2.1. Participants

This study was conducted on female patients aged 60 to 70 years old who visited C Hospital in Seoul, Korea, for chronic low back pain. The inclusive criteria for the study were as follows: active participation in the study, sufficient understanding of the study, completion of the study consent form, voluntary participation in the study, and a diagnosis of nonspecific chronic low back pain related to neurological abnormalities by the doctor in charge based on radiographic examination. Those who had a prevalence period of at least 3 months after the diagnosis were also considered.

The exclusion criteria for the study were as follows: motor and sensory dysfunction, neurological abnormalities, muscle paralysis, doctor’s orders to refrain from exercising, limited range of motion due to acute lumbar pain, and mental problems or inability to understand the study. Patients with difficulty in participating correctly, those with dizziness or hypertension (blood pressure of ≥160/110 mmHg), and those who could not take the basic posture of LSEs because of pain were included.

The present study was approved by the institutional review board of Sahmyook University (Seoul, Korea, 2-1040781-A-N-012020081HR) and it was registered (KCT0005499) in the Clinical Research Information Service of the Republic of Korea. The objective and procedures of the study were fully understood by the participants, and all participants provided informed consent for inclusion. This study was performed in accordance with the ethical principles of the Declaration of Helsinki.

### 2.2. Experimental Procedures

We used G*Power (version 3.1.9.7; Franz Faul, Kiel, Germany, 2020) for power analysis before recruiting participants. The overall effect size index for all outcome measures and the power of the study were 0.5 and a probability of 0.05 to minimize type II error (80% power), respectively. The estimated target sample size was 34; therefore, we recruited 40 low back pain patients for this experiment.

The 40 participants who met the selection conditions provided consent after receiving detailed explanations of the experimental procedure. After the pre-test, all participants were randomly divided into two groups which performed PNE with LSEs (*n* = 20) or the control group (*n* = 20), which performed LSEs alone using the Research Randomizer program (http://www.randomizer.org/, accessed on 25 September 2020). To blind participants to group allocation, they were only informed about the general description of the study design. However, they were not informed about the type of intervention. All tests were measured by physical therapists who were not involved in this study, and to minimize the measurement error, the same examiner performed the measurements before training and after 8 weeks of training.

All participants were evaluated for strength, pain, flexibility, and activity disorder index before and after training for 8 weeks. Muscle strength was measured by raising the upper body through a sit-up motion or tilting the upper body backward in the prone posture and holding the positions. Pain was measured using the Numerical Pain Rating Scale (NPRS), Korean Pain Catastrophizing Scale (K-PCS), and Tampa Scale of Kinesiophobia-11 (TSK-11) through questionnaires. Flexibility was measured using the finger-to-floor test (FFT) and modified–modified Schober’s test (MMST). The activity disorder index was evaluated using the Roland–Morris Disability Questionnaire (RMDQ).

In both groups, the 8-week-long intervention was performed once a day for a total of 50 min, twice a week. Furthermore, the experimental and control groups underwent the same physical therapy (hyperthermia, electrotherapy) for 20 min. Additionally, the experimental group was administered PNE before each intervention. After 10 min of PNE and physical therapy, 20 min of LSEs were performed. Meanwhile, the control group performed only 30 min of LSEs after physical therapy. Two and three participants dropped out of the experimental group and the control group, respectively. A total of 35 participants, excluding the 5 who dropped out, completed the study.

**Figure 1:**
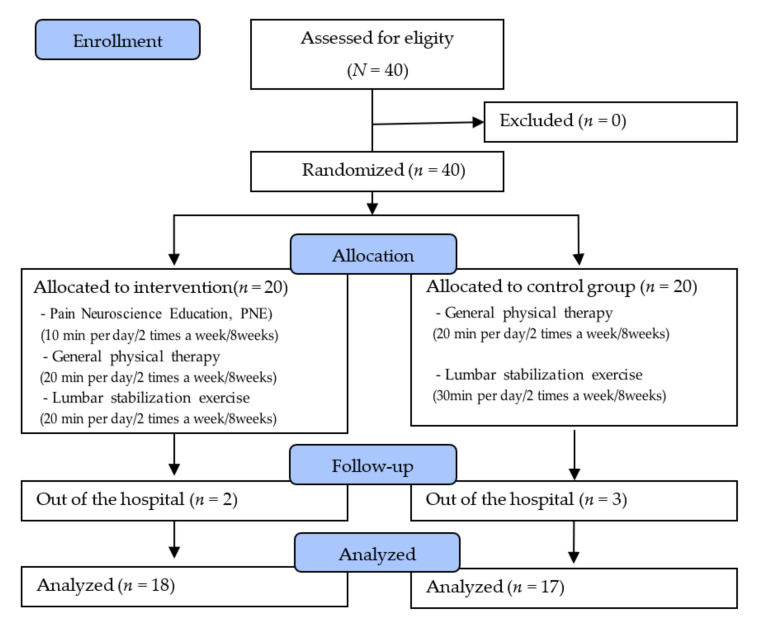
Flow diagram of total experimental procedure.

The physical therapists who participated in the study had been trained for the LSEs that were applied to the patients for >3 years in advance and were thus fully aware of possible problems. The experiments were always conducted by the same therapist. The same researcher conducted PNE before the start of the experiment (Figure 1).

#### 2.2.1. Pain Neuroscience Education

The PNE used in this study was aimed at lowering the threat value of pain, increasing the participants’ knowledge of pain, and reconceptualizing pain. To achieve these aims, the participants needed to understand that all pain is created, composed, and controlled by the brain, and that pain symptoms are often associated with hypersensitivity of the central nervous system and not with tissue damage [28]. Excerpts from the book Explain Pain [29] were taken and verbally explained to the participants [23,30,31]. The education consisted of a total of eight topics, with oral explanations provided with presentation materials. The education was delivered to participants by the physical therapist who had completed a PNE class and had more than five years of clinical experience. During training, all major concepts of pain neurophysiology were explained and discussed [32]. PNE training was conducted twice a week for 10 min before the start of all treatments (Table 1).

#### 2.2.2. LSE Training 

LSE training was performed for 20–30 min per session, once a day, twice a week, depending on the group. The difficulty was gradually increased from low to high. The LSE intervention used in this study consisted of 11 exercise methods aimed at strengthening the lumbar stabilization muscles such as the transversus abdominis, multifidus, and oblique abdominal muscles. In addition, detailed explanations were provided to the participants [33] by the physical therapist in charge, who had learned and trained with the postures for ≥3 years (Table 2).

### 2.3. Outcome Measurements

#### 2.3.1. Primary Outcome: Muscle Strength

Muscle strength was measured using sit-up and back-up motions. The same physical therapist used a stopwatch to measure the retention time of the abdominal and back muscles twice, and the average values were recorded in seconds. Muscle strength measurements included abdominal muscle strength, back muscle strength, and muscle endurance. The muscle strength of the abdomen and back was measured using the method of holding sit-ups and tilting the back while in the prone position, respectively.

First, abdominal muscle strength was measured in seconds by raising the upper body and holding the position. The participants lied on a mat, with the knees bent at 90 and the arms crossed on the chest. The upper body was lifted by sitting up, the position was held, and the elapsed time was measured. Second, the back muscle strength was measured in seconds by leaning the upper body backward and holding the position. The participants were positioned prone with both hands on the back of the waist. The back was lifted, the back-up position was held, and the elapsed time was recorded.

#### 2.3.2. Primary Outcome: Pain

Pain was measured using the NPRS, K-PCS, and TSK-11.

The NPRS is a self-report measurement tool that can be completed in <1 min. A score range of 0–10 is set on the horizontal line, with 0 points on the left and 10 points on the right, indicating the worst imaginable excruciating pain [34,35,36]. The NPRS is a simple and highly reproducible method of expressing the degree of pain, with a high sensitivity, interrater reliability of 0.90 [37], and test–retest reliability of 0.95–0.96 [38]. In this study, the reliability of the tool was 0.95 (Cronbach’s alpha coefficient).

The K-PCS is the Korean version of the Pain Catastrophizing Scale (PCS) developed by Sullivan et al. (1995) [39], which was modified and supplemented by Cho et al. [40]. The K-PCS consists of 13 questions, with three subfactors: ruminant thinking, hyperbolic thinking, and helplessness [39]. On a Likert five-point scale, it is evaluated by summing the scores, ranging from 0 point (not at all) to 4 points (always yes), with the total score ranging from 0 to 52 points. The higher the total score, the higher the degree of negative and exaggerated thoughts related to pain. The clinical judgment criteria were based on several preceding studies, with a total score of 0–9 being low, 10–19 intermediate, 20–39 high, and 40–52 very high. The reliability of the tool was a Cronbach’s α of 0.93 in the study by Cho et al. (2013). In this study, the reliability of the tool was a Cronbach’s alpha coefficient of 0.84.

The TSK was devised as 17 items by Miller et al. (1991) [41], and Tkachuk et al. (2012) [42] modified the tool to 11 items (TSK-11). The TSK-11 is composed of 11 items within two factors (pathological somatic focus and activity avoidance) and is scored on a four-point scale (from 1 to 4) for each item. The score range is 11–44 points, in which the higher the score, the higher the level of motor fear [42]. The internal consistency of the TSK-11 is high, with a Cronbach’s α coefficient of 0.80, and the reliability is moderate. The TSK-11 is a simple, reliable, and valid tool for measuring fear of movement and (re)injury in patients with chronic pain. The internal agreement in this study was 0.86 [42].

#### 2.3.3. Secondary Outcome: Flexibility

Flexibility was measured using the MMST and FFT. The measurements were performed three times by the same physical therapist, and the average value was used.

The MMST is a method of measuring lumbar flexion to assess flexibility. This test can separate and measure movements in the lumbar area using a tape. In the test method, with the participant in an upright position, the examiner marked two landmarks with a black pen: one landmark 5 cm below the midpoint of both sides of the participant’s posterior superior iliac spine line and another point 10 cm above the line. The participant made maximum effort to touch the floor, with the knees, elbows, and fingers open. In this state, the distance between the two landmarks was measured with a tape measure and recorded. The longer the distance, the better the flexibility. The validity and reliability were r = 0.67 and intraclass correlation coefficient (ICC) = 0.91, respectively [43].

In the FFT, the participants stood on a flat box without shoes, with the big toes of both feet not crossing the corners of the box. The participants bent to reach the toes, and the distance between the floor and fingers was measured. The upper-body forward lean test has high validity (RS = −0.96) and high inter-rater and intra-rater reliability (ICC = 0.99) [44].

#### 2.3.4. Secondary Outcome: Activity Disorder Index

The activity disorder index was assessed using the RMDQ, which measures the degree of dysfunction caused by low back pain. It consists of a 0- to 24-point scale and is a commonly used assessment method for low back pain disability parameters, such as the Oswestry Disability Index, in clinical settings. The reliability (ICC) of the RMDQ is very high at 0.932, and the Korean version of the RMDQ, which was proven reliable by Lee et al., was used in this study [45].

### 2.4. Statistical Analysis

SPSS statistical software (version 22.0; IBM, Chicago, IL, USA) was used for all statistical analyses. The Shapiro–Wilk test was used to analyze the normal distribution of the variables. The independent-samples *t*-test was performed to identify differences between the groups. The paired t-test was used to compare the results before and after the intervention. Lastly, the analysis of covariance was performed to identify differences between the groups. The level of statistical significance was set at *p* < 0.05.

## 3. Results

We included more than 80% compliance to treatment for statistical analysis and used data from 35 patients because 5 out of 40 dropped out due to personal reasons. The demographic characteristics are shown in Table 3. No significant differences were observed in the baseline values between the PNE plus LSE group and the LSE group for all parameters.

### 3.1. Muscle Strength

The results of the comparison of pre-/post-test measures of the strength of the abdominal and back muscles between the two groups are shown in Table 4. The strength of the abdominal and back muscles significantly improved after the intervention within the PNE plus LSE group and the LSE group (*p* < 0.05), with a significant difference between the groups (*p* < 0.05). Covariance analysis was conducted to determine whether there was a difference between the groups in terms of the level of change in the abdominal and back muscles after intervention. The muscle strength was processed as a covariate (Table 4). The results of the study showed statistically significant differences in the abdominal muscle strength (F = 9.598; *p* = 0.005), and back muscle strength (F = 7.102; *p* = 0.014). These results indicate that the PNE plus LSE group had a statistically significant improvement in the muscle strength compared to that in the LSE group, demonstrating that the training program in this study was effective.

### 3.2. Pain

The results of comparison of pre-/post-test measures of pain in the NPRS, K-PCS, and TSK-11 between two groups are shown in Table 5. Pain evaluated using the NPRS, K-PCS, and TSK-11 significantly improved after the intervention within the PNE plus LSE group and the LSE group (*p* < 0.001), and also showed significant differences between the two groups (*p* < 0.05). Covariance analysis was conducted to determine whether there was a difference between the groups in terms of the level of change in the pain after intervention. The pain was processed as a covariate. The results of the study showed statistically significant differences in the NPRS (F = 24.286; *p* < 0.001), K-PCS (F = 11.558; *p* = 0.003), and TSK-11 (F = 13.179; *p* = 0.001). These results indicate that the PNE plus LSE group had a statistically significant improvement in the pain compared to that in the LSE group, demonstrating that the training program in this study was effective. 

### 3.3. Flexibility

The results of the comparison of pre-/post-test measures of flexibility in FFT and MMST between the two groups are shown in Table 6. The flexibility significantly improved after the intervention within the PNE plus LSE group and the LSE group (*p* < 0.05). Covariance analysis was conducted to determine whether there was a difference between the groups in terms of the level of change in the flexibility after intervention. The flexibility was processed as a covariate. The results of the study showed statistically significant differences in the FFT (F = 4.327; *p* = 0.049), but no significant difference in MMST (F = 3.451; *p* = 0.077).

### 3.4. Activity Disorder

The results of comparison of pre-/post-test measures of activity disorder in the RMDQ between the two groups are shown in Table 7. The RMDQ score significantly improved after the intervention within the PNE plus LSE group and the LSE group (*p* < 0.05). 

Covariance analysis was conducted to determine whether there was a difference between the groups in terms of the level of change in the activity disorder after intervention. The activity disorder was processed as a covariate. The results of the study showed statistically no significant differences in the RMDQ score (F = 0.081; *p* = 0.778). 

## 4. Discussion

This study found the effects of PNE combined LSEs on muscle strength and pain in female patients for chronic low back pain.

In the comparison of muscle strength between before and after the intervention in this study, the abdominal muscle strength of the experimental group increased from 33.83 to 41.33 s, and the back strength increased from 16.28 to 26.00 s, with significant differences (*p* < 0.05). In the comparison of the difference in the amount of change between the two groups, the experimental group showed a significantly higher difference in both abdominal strength and back strength than the control group (*p* < 0.05), indicating that the combination of PNE and LSEs is more effective in improving muscle strength in patients with chronic low back pain. 

In a study by Rosa Andias et al. (2018) [46], muscle endurance was significantly improved before and after the experiment in the experimental group (before and after difference, +47.5 s) and in the control group (before and after difference, +14.2 s). The comparison between the two groups also showed a significant difference. It has been shown that neuroscience education is effective in improving muscle strength. In this study, PNE increased the patients’ interest in functional activity and exercise and motivated them to perform more frequent and intense activities, thereby improving physical ability and reducing pain [47]. In addition, PNE has been reported to have a positive effect of changing incorrect pain beliefs and incorrect movements of the lower back [23], thus allowing patients to more actively participate in the LSE program without fear of exercise. The LSE intervention time of the experimental group was 10 min shorter than that of the control group; however, the lumbar muscle strength was further increased because of the additional exercise performed at home.

In the comparison of the change in pain before and after the intervention in this study, the pain score (NPRS) of the experimental group ranged from 4.67 to 2.78 points. Although we achieved statistical significance between groups, this does not mean clinical meaningful results. The minimum clinically important difference (MCID) of NPRS for low back pain is a 2 point change [48]. The K-PCS score decreased from 20.06 to 12.17 points, and the TSK-11 score decreased from 54.92% to 38.13%, with a significant difference between before and after the experiment (*p* < 0.05). In the comparison of the difference in the amount of change between the two groups, the experimental group showed significantly better improvement than the control group in terms of pain score, catastrophic level of pain, and level of motor fear (*p* < 0.05). The PCS score’s minimal detectable change (MDC) was reported to be 9.1 points for low back pain, and our results did not reach a clinically meaningful difference [49].

In a study by Pires et al. (2015) [50], 62 patients with chronic low back pain were divided into 30 participants in the experimental group who underwent PNE and aquatic exercise training and 32 participants who performed water exercises alone, with 12 aquatic exercise programs applied for 6 weeks. In the case of the experimental group, two PNE programs were applied before the aquatic exercises. The pain score, motor fear level, and dysfunction before and after the experiment were measured at three time points: before, 6 weeks after, and 3 months after the experiment. The pain score of the experimental group changed from 43.4 to 20.6 points after 6 weeks and to 18.0 points after 3 months. In the case of the control group, the score decreased from 42.4 to 27.6 points after 6 weeks and then increased again to 35.8 points after 3 months. In the case of motor fear level, the score in the experimental group decreased from 28.6 to 25.2 points after 6 weeks and to 23.2 points after 3 months. In the control group, the score decreased from 29.1 to 27.5 points after 6 weeks and to 26.5 points after 3 months. As a result, only the pain score showed statistical significance in the comparison between groups.

The PNE used in this study changes misconceptions or coping strategies for pain and disease, thereby improving pain and showing a positive effect on learning normal movements and activities [51]. It is believed that such an education has a positive effect on pain improvement. However, previous research has shown that although PNE is effective in reducing pain when combined with an exercise program, as observed in this study, pain may increase again after a period if education is not provided. Therefore, the number of training sessions might also have an effect on the therapeutic outcome and can have a positive effect on long-term pain improvement.

In a study by Malfliet et al. (2018), 120 patients with nonspecific chronic spinal pain were divided into an experimental group that received PNE and a control group that received education about the biological properties of the back and neck. The levels of motor fear and catastrophic pain were evaluated before and after training. As a result, the level of motor fear decreased from 34.37 to 30.32 points in the experimental group and from 36.72 to 35.73 points in the control group, with a statistically significant difference within and between groups, indicating that PNE training was more effective (*p* < 0.05). Woby et al. (2004) reported that pain reconceptualization through PNE decreases the threshold for pain and changes incorrect beliefs and thoughts about the causes of pain [52]. Therefore, previous studies indicate that although immediate and positive effects appear after only a short period of education, continuing such education may have considerable long-term effects. More studies should be conducted in the future to determine the number of sessions and the duration of education that can show the most appropriate and the greatest effect, in order to establish the most efficient treatment method.

With respect to the change in flexibility before and after the intervention in this study, the FFT result in the experimental group increased from −1.83 cm (before the experiment) to −1.16 cm (after the experiment). The MMST result increased from 3.51 to 3.71 cm in the experimental group. A significant difference was observed in the FFT and MMST within groups (*p* < 0.05), but the FFT only showed a significant difference between groups (*p* < 0.05). These results do not reveal the clinical significance. In a future study, a systematic and balanced exercise program that can improve flexibility as well as muscle strength and pain should be applied, and the component involving exercising at home should be monitored daily to provide motivation. We believe that better research results will emerge if this can be efficiently implemented.

The changes in the activity disorder index before and after the intervention in this study were evaluated using the RMDQ. As a result, the activity disorder index in the experimental group decreased from 9.94 to 6.06 points, showing significant differences between before and after the experiment (*p* < 0.05). However, no significant differences between the two groups were observed, and thus it is unknown whether the combination of PNE and LSEs has a positive effect on activity disorder index in patients with chronic low back pain. Moseley et al. (2004) divided a total of 58 patients with chronic low back pain into 31 participants in the experimental group who received PNE and 27 control participants who received education on the anatomy and physiology of the back. The activity disorder index was evaluated using the RMDQ before and after the experiment. As a result, the activity disorder index of the experimental group decreased from 15 to 14 points, whereas that of the control group increased from 15 to 16 points, with a statistically significant difference (*p* < 0.05). Although our result showed no statistical significance between the two groups in RMDQ, we achieved a clinically significant MCID of RMDQ i.e., the recommended 2.5 points [53].

This study had some limitations. First, there is a limit to generalizing our results, as the number of participants was small, the patient’s clinical profile, such as risk factors or comorbidities was a limited reflection, and the data were limited to one hospital in Seoul. Second, as the frequency of confirming the effect was low (twice a week), the total intervention time was somewhat insufficient relative to the study period. Third, in providing PNE, we attempted to use supplemental materials such as pictures or images to improve the ease of understanding of the theoretical contents for older patients; however, there was some difficulty. Therefore, future studies will need to not only develop educational programs using simpler words and various data that general patients can easily understand, but also to include more participants. In addition, in the process of performing LSEs, there was a difference in the participants’ understanding of the exercise. In the first week, the time for re-education about individual muscles and the principles of exercise accounted for >50% of the exercise time. Therefore, in future studies, it should be considered that participants with chronic low back pain may have lower awareness of posture or movements than normal participants, and it is necessary to ensure sufficient understanding of the participants before conducting the program.

## 5. Conclusions

This study proved that the combination of the pain neuroscience education combined with lumbar stabilization exercises is more effective than lumbar stabilization exercises alone as a treatment method for improving muscle strength and reducing pain in female patients with chronic low back pain. However, further studies on various educational and exercise programs with large samples are needed.

## Figures and Tables

**Table 1:** Pain neuroscience education topics.

	Weekly Training Topics (Training Time: 10 min Each)
1. Neurophysiology of pain	Explain the basic theory of the structure of the brain and nerves, the peripheral nervous system, and the central nervous system by comparing to a mobile phone or computer.
2. Nociception	Explain and compare concepts and differences between acute and chronic pain with real-world experiences and emphasize the importance of exercise.
3. Nociceptive pathways	Explains the paths of pain sensation and noxious/innoxious pain sensations, and highlights how psychological aspects and thoughts affect pain.
4. Neurons and synapses	Explain the structure and function of neurons and synapses, the processes of electrical signals, and how the patient’s thoughts can influence these signaling processes.
5. Action potential	Describe action potentials and thresholds, and differences in sensations felt by each person by comparing them to signals and alarms generated by the body.
6. Spinal inhibition and facilitation	Explain the process of inhibition and promotion of the spine by comparing the process of electrical signal transmission and homeostasis and emphasize the change in sensation according to the state of the body.
7. Sensitization	Explain the types of sensations, the process of transmitting each sensation, and the differences, and emphasize the understanding of pain through the gate control theory.
8. Plasticity of the nervous system	Explain the basic concept of neuroplasticity in relation to changes in the brain caused by experience and learning and emphasize positive changes through exercise.

The “How-to” of Teaching Patients About Pain.

**Table 2:** Lumbar stabilization exercise on the mat.

Item	Training Method
Hamstring stretching	In the supine position, place a towel under the sole of the foot, hold the ends of the towel with both hands, extend the knee, lift the leg, and repeat the stretching five times.
Abdominal stretching	In the prone position, place both hands on the floor, stretch the elbows and lift the upper body, and repeat the abdominal muscle stretching five times.
Quadriceps stretching	In the lying position, bend one knee, pull back the lower leg with the arm of the same side, and repeat the stretching five times.
Cat–camel	In the crawling position, contract the abdominal muscles and bend and open the back five times.
Neutral position	In the knee-bent and lying position, pull the abdominal muscles to contract the transverse abdominal muscles and the pelvic floor muscles isometrically, and maintain for 5 s. Repeat five times.
Curl up	In the knee-bent and lying position, up the trunk to contract the transverse abdominal muscles and maintain for 5 s. (arm position: knee-chest-head) Repeat five times.
Dead bug	In the supine position, bend hip and knee 90 degree and lift from floor, flex the opposite shoulder and maintain for 5 s. Repeat five times.
Side bridge	In the side lying position with knee flexion, left the pelvic off from the floor and maintain for 5 s. Repeat five times.
Superman	In the prone position with arms straight overhead and legs fully extended, simultaneously lift arms and legs off the floor and maintain for 5 s. Repeat five times.
Bridge	In the supine position with knee flexion, left the hips off from the floor and maintain for 5 s. Repeat five times.
Quadruped position with lifting arm and leg	In the quadruped position, left the arm and leg off from the floor and maintain for 5 s. Repeat five times.

5 repetitions/1 set, rest time sets 20 s. Additional rest time was allowed when fatigue occurred or on a patient’s request.

**Table 3:** Demographic data of the two groups (*N* = 35).

Parameters	PNE + LSE (*n* = 18)	LSE (*n* = 17)	t/x^2^ (*p*)
Age (years)	68.89 (5.08)	71.29 (5.18)	−1.386 (0.175)
Height (cm)	159.94 (3.42)	161.94 (3.63)	−1.675 (0.103)
Weight (kg)	58.22 (2.94)	60.65 (6.09)	−1.486 (0.151)
Obesity rate (%)	22.76 (0.94)	23.10 (1.86)	−0.677 (0.505)
Duration of injury (months)	18.50 (6.767)	19.71 (5.871)	0.562 (0.578)
radiating pain (Yes/No)	3/15	2/15	0.172 (0.679)
Analgesic use (Yes/No)	4/14	3/14	0.114 (0.735)
Hypertension (Yes/No)	11/7	10/7	0.19 (0.890)
Hypercholesterolemia (Yes/No)	6/12	7/12	0.230 (0.631)
Previous history of cardiovascular disease (Yes/No)	4/14	4/13	0.008 (0.927)

Data are mean (standard deviation). PNE, pain neuroscience education; LSE, lumbar stabilization exercise.

**Table 4:** Comparison of muscle strength within groups and between groups (*N* = 35).

Parameters		Pre-Test	Post-Test	Group Difference, Mean (95% CI)	SS	df	MS	Effect Size	t(*p*)/F(*p*)
Abdominal muscle strength (sec)	PNE + LSE	33.83 (5.29)	41.33 (4.67)	−7.500 (−9.111 to –5.889)					−9.820 (0.000)
	LSE	31.71 (4.78)	36.94 (4.25)	−5.235 (−5.904 to –4.567)					−16.599 (0.000)
	Covariate				716.596	12	59.716		11.626 (0.000)
	Group				49.302	1	49.302	0.304	9.598 (0.005)
	Error				113.004	22	5.137		
Back muscle strength (sec)	PNE + LSE	16.28 (1.93)	26.00 (2.22)	−9.722 (−10.877 to –8.568)					−17.769 (0.000)
	LSE	15.76 (1.95)	23.18 (3.50)	−7.412 (−9.007 to –5.816)					−9.846 (0.000)
	Covariate				189.558	12	15.796		2.1642 (0.056)
	Group				51.851	1	51.851	0.244	7.102 (0.014)
	Error				160.614	22	7.301		

Data are mean (standard deviation). PNE, pain neuroscience education; LSE, lumbar stabilization exercise; *p* < 0.05.

**Table 5:** Comparison of pain within groups and between groups (*N* = 35).

Parameters		Pre-Test	Post-Test	Group Difference, Mean (95% CI)	SS	df	MS	Effect Size	t(*p*)/F(*p*)
NPRS (points)	PNE + LSE	4.67 (1.45)	2.78 (1.26)	1.89 (1.65 to 2.12)					17.000 (0.000)
	LSE	4.59 (1.66)	3.47 (1.37)	1.12 (0.87 to 1.37)					9.500 (0.000)
	Covariate				57.700	12	4.808		27.524 (0.000)
	Group				4.243	1	4.243	0.525	24.286 (0.000)
	Error				3.843	22	0.175		
K-PCS (points)	PNE + LSE	20.06 (2.53)	12.17 (2.61)	7.89 (7.02 to 8.76)					19.178 (0.000)
	LSE	18.94 (2.56)	13.47 (2.76)	5.47 (4.81 to 6.13)					17.615 (0.000)
	Covariate				197.000	12	16.417		6.381 (0.000)
	Group				29.736	1	29.736	0.344	11.558 (0.003)
	Error				56.600	22	2.573		
TSK-11 (%)	PNE + LSE	54.92 (9.93)	38.13 (7.89)	16.79 (13.99 to 19.59)					12.655 (0.000)
	LSE	56.95 (6.93)	45.72 (7.31)	11.23 (8.70 to 13.76)					9.414 (0.000)
	Covariate				1949.755	12	162.480		7.614 (0.000)
	Group				281.246	1	281.246	0.375	13.179 (0.001)
	Error				469.497	22	21.341		

Data are mean (standard deviation). PNE, pain neuroscience education; LSE, lumbar stabilization exercise; NPRS, Numerical Pain Rating Scale; K-PCS, Korean Pain Catastrophizing Scale; TSK-11, Tampa Scale of Kinesiophobia-11; *p* < 0.05.

**Table 6:** Comparison of flexibility within groups and between groups (*N* = 35).

Parameters		Pre-Test	Post-Test	Group Difference, Mean (95% CI)	SS	df	MS	Effect Size	t(*p*)/F(*p*)
FFT (cm)	PNE + LSE	−1.83 (3.73)	−1.16 (3.91)	−0.66 (−0.99 to −0.33)					−4.225 (0.001)
	LSE	0.17 (3.52)	0.58 (3.51)	−0.41 (−0.72 to −0.10)					−2.820 (0.012)
	Covariate				476.447	12	39.704		89.906 (0.000)
	Group				1.911	1	1.911	0.164	4.327 (0.049)
	Error				9.716	22	0.442		
MMST (cm)	PNE + LSE	3.51 (0.78)	3.71 (0.74)	−0.20 (−0.27 to –0.12)					−5.532 (0.000)
	LSE	3.23 (0.84)	3.37 (0.83)	−0.14 (−0.19 to −0.08)					−5.470 (0.000)
	Covariate				22.141	12	1.845		126.527 (0.000)
	Group				0.050	1	0.050	0.077	3.451 (0.077)
	Error				0.31	22	0.015		

Data are mean (standard deviation). PNE, pain neuroscience education; LSE, lumbar stabilization exercise; FFT, finger-to-floor test; MMST, modified–modified Schober’s test; *p* < 0.05.

**Table 7:** Comparison of activity disorder within groups and between groups (*N* = 35).

Parameters		Pre-Test	Post-Test	Group Difference, Mean (95% CI)	SS	df	MS	Effect Size	t(*p*)/F(*p*)
RMDQ (points)	PNE + LSE	9.94 (1.58)	6.06 (1.79)	3.89 (3.13 to 4.65)					10.786 (0.000)
	LSE	10.94 (1.56)	7.18 (1.97)	3.76 (3.23 to 4.29)					15.033 (0.000)
	Covariate				80.019	12	6.668		3.032 (0.012)
	Group				0.179	1	0.179	0.004	0.081 (0.778)
	Error				48.381	22	2.199		

Data are mean (standard deviation). PNE, pain neuroscience education; LSE, lumbar stabilization exercise; RMDQ, Roland–Morris disability questionnaire; *p* < 0.05.

## Data Availability

Not applicable.
